# Evaluation of Various Support Intensities of Digital Mental Health Treatment for Reducing Anxiety and Depression in Adults: Protocol for a Mixed Methods, Adaptive, Randomized Clinical Trial

**DOI:** 10.2196/45040

**Published:** 2023-04-28

**Authors:** Brooke Andrews, Britt Klein, Suzanne McLaren, Shaun Watson, Denise Corboy

**Affiliations:** 1 Health Innovation & Transformation Centre Federation University Australia Ballarat Australia; 2 Biopsychosocial & eHealth Research & Innovation Federation University Australia Ballarat Australia; 3 Charles Sturt University Port Macquarie Australia; 4 Blue Sky Mind Research Consultancy Ballarat Australia

**Keywords:** video chat therapy, therapist assistance, self-help, transdiagnostic, digital intervention, anxiety, depression, comorbidity

## Abstract

**Background:**

Anxiety and depression are leading causes of disease worldwide, requiring timely access to evidence-based treatment. Digital mental health (dMH) interventions increase accessibility to evidence-based psychological services delivered in a variety of web-based formats (eg, self-help and therapist-assisted interventions). Robust and rigorous studies of adaptive web-based intervention designs are scarce. No identified randomized clinical trial has investigated the efficacy of a 2-stage adaptive design, whereby the program-only condition or *no support* dMH treatment program is augmented by either low or high therapist assistance, if a participant does not improve or engage in the program-only condition.

**Objective:**

The primary objective is to assess whether low or high therapist-assisted support delivered via video chat is more effective in reducing anxiety and depressive symptoms compared with a dMH program–only condition. The secondary objective is to evaluate the role of motivation; self-efficacy; and preferences in participant engagement, adherence, and clinical outcomes (anxiety and depression symptoms) among the 3 treatment conditions (program only, low-intensity therapist assistance, and high-intensity therapist assistance). A mixed methods analysis of factors affecting participant attrition, participant reasons for nonengagement and withdrawal, and therapist training and implementation of dMH interventions will be completed. Qualitative data regarding participant and therapist experiences and satisfaction with video chat assessment and treatment will also be analyzed.

**Methods:**

Australian adults (N=137) with symptoms or a diagnosis of anxiety or depression will be screened for eligibility and given access to the 8-module Life Flex dMH treatment program. On day 15, participants who meet the augmentation criteria will be *stepped up* via block randomization to receive therapist assistance delivered via video chat for either 10 minutes (low intensity) or 50 minutes (high intensity) per week. This adaptive trial will implement a mixed methods design, with outcomes assessed before the intervention (week 0), during the intervention (weeks 3 and 6), after the intervention (week 9), and at the 3-month follow-up (week 21).

**Results:**

The primary outcome measures are for anxiety (Generalized Anxiety Disorder–7) and depression severity (Patient Health Questionnaire–9). Measures of working alliance, health status, health resources, preferences, self-efficacy, and motivation will be used for secondary outcomes. Qualitative methods will be used to explore participant and therapist experiences of video chat assessment and treatment, participant reasons for withdrawal and nonengagement, and therapist training and implementation experiences. Data collection commenced in November 2020 and was completed at the end of March 2022.

**Conclusions:**

This is the first mixed methods adaptive trial to explore the comparative efficacy of different intensity levels of self-help and a therapist-assisted dMH intervention program delivered via video chat for adults with anxiety or depression. Anticipated results may have implications for the implementation of dMH interventions.

**Trial Registration:**

Australian and New Zealand Clinical Trials Registry 12620000422921; https://tinyurl.com/t9cyu372

**International Registered Report Identifier (IRRID):**

RR1-10.2196/45040

## Introduction

### Background

The Global Burden of Diseases, Injuries, and Risk Factors Study 2019 identified depression and anxiety disorders as the 2 most debilitating mental disorders worldwide [1]. A recent study by Collaborators Cochrane Mental Disorders [2] quantified the global impact of COVID-19 on the prevalence of depression and anxiety disorders. That study estimated, for depression, an international prevalence of 246 million people (increase from 193 million people before the pandemic) and, for anxiety disorders, a prevalence of 374 million people internationally (increase from 298 million people before the pandemic). Such statistics demonstrate that anxiety and depressive disorders are leading diseases on a global scale that become more prevalent during global events such as the COVID-19 pandemic.

Rates of help seeking for a mental health–related service have also increased in light of the COVID-19 pandemic [3]. There has been increased use of digital mental health (dMH) interventions and telehealth services, attributable first to their ability to disseminate and scale quickly and, second, to their utility as an adjunct to existing face-to-face mental health services [4]. However, long wait lists to access psychological treatment via telehealth are reported [5,6], as are substantial costs associated with the delivery of psychological services via telehealth [3].

To ensure access to evidence-based treatment for the wider community, empirical research conducted over the past 20 years has investigated different methods of delivering psychological treatment. As an alternative to the standard in-person, face-to-face model, dMH interventions now have a well-established evidence base for the provision of psychological treatment for anxiety and depression delivered via the internet, in a variety of formats (eg, self-help and therapist-assisted interventions), mitigating the increasing demands for mental health services.

The dMH interventions serve to increase accessibility to evidence-based services within a high-volume, low-intensity treatment model [7]. Low-intensity treatment models have been developed to assist individuals experiencing mild to moderate psychological difficulties and propose that more intensive treatment regimens could then be provided to individuals who do not benefit from less intensive forms of psychological treatment [8]. The dMH interventions can address the evolving needs of consumers by adapting the implementation of treatment such that individuals can be *stepped up* to receive high-intensity treatment if required.

Adaptive intervention treatment designs use decision rules to determine whether and how the delivery of a course of treatment is altered [9]. Stepped care models are identified as a type of adaptive intervention design. Stepped care models incorporate a low-intensity intervention that is increased if certain criteria are fulfilled and can often include augmenting, switching, or maintaining a course of treatment based on some type of consumer characteristic (ie, low engagement levels) or response (ie, symptom severity) [9]. Within a stepped care model, dMH interventions are offered as part of the first step of self-management (eg, self-help web-based program), and if required, an individual’s treatment can be increased, whereby a therapist is added to support the dMH intervention.

Previous empirical studies investigating the effectiveness of therapist-assisted dMH interventions for anxiety and depression have largely used telephone, instant messaging, and email assistance. Recent advances in dMH include the use of video chat technology, in which face-to-face synchronous digital communication most closely aligns with the traditional in-person, gold-standard service delivery model in psychology. Empirical investigation into the efficacy of video chat, compared with that of other dMH care models for treating anxiety and depression, is lacking. Most of the research studies completed so far have investigated posttraumatic stress disorder and have compared psychiatric treatment delivered via video chat with that delivered in-person, with comparable outcomes reported [10,11].

Currently, there is a paucity of robust and rigorous studies on the use of video chat methods in dMH interventions. No identified clinical trial has investigated the efficacy of a 2-stage adaptive design offering the *no therapist support* dMH program and augmenting a participant’s program with either low or high therapist assistance if they do not improve or engage in the *no therapist support* program-only condition. Within the field of psychology, the literature about dMH is also largely biased toward quantitative research methodology that minimizes the opportunity to more closely investigate factors contributing to the efficacy of various intensities of a dMH treatment program (eg, participant attrition and engagement factors). Therefore, the testing of an adaptive, mixed methods randomized clinical trial design will provide great insight into participant characteristics of engagement; efficacy of video chat as a mode of therapist assistance in dMH interventions; and, overall, improvement of future services delivered via dMH.

### Objectives

The purpose of this study is to present a protocol designed to evaluate various support intensities of a dMH treatment program for anxiety and depression within a mixed methods design. Therefore, the study has the following primary objectives:

1. To compare the efficacy of clinical outcomes (anxiety [Generalized Anxiety Disorder–7; GAD-7] and depression [Patient Health Questionnaire–9; PHQ-9] symptoms) among the 3 treatment conditions (dMH program only, low-intensity therapist assistance, and high-intensity therapist assistance), with particular focus on whether therapist-assisted support produces greater reductions in anxiety and depression severity compared with those produced by dMH program–only support.
2. To examine the role of motivation and self-efficacy on engagement, adherence, and clinical outcomes (anxiety [GAD-7] and depression [PHQ-9] symptoms) among the 3 treatment conditions (dMH program only, low-intensity therapist assistance, and high-intensity therapist assistance) at postintervention (week 9) and follow-up (week 21) assessments, compared with those at preintervention (week 0) assessment.
3. To explore participant and therapist experiences and satisfaction with assessment and therapist assistance delivered via video chat technology, using semistructured interviews; specific areas of focus will include the ability to develop and maintain therapeutic relationships, implementation barriers and facilitators, and identified strengths and limitations of video chat technology for conducting assessments and delivering dMH treatment.
4. To explore therapist training and implementation experiences in the delivery of dMH interventions.
5. To use a mixed methods analysis to examine factors affecting participant attrition in each of the treatment intensities; quantitative examination of preferences, treatment condition allocation (dMH program only, low-intensity therapist assistance, and high-intensity therapist assistance), and attrition rates will be explored; qualitative exploration of participant reasons for withdrawal and nonengagement with therapist support will also be conducted using semistructured interviews.
6. To explore the outcomes of working alliance, health resources, and health status among the 3 treatment conditions (dMH program only, low-intensity therapist assistance, and high-intensity therapist assistance).

## Methods

### Trial Design

This trial will follow the International CONSORT (Consolidated Standards of Reporting Trials) guidelines and evaluate the conditions using an adaptive randomized clinical trial design [12]. The clinical trial also adheres to SPIRIT (Standard Protocol Items: Recommendations for Interventional Trials; Multimedia Appendix 1). All information will be strictly confidential, yet subject to legal limitations (eg, legal subpoena), and only accessed by the investigators. Data are stored securely using 256-bit encryption Secure Sockets Layer and Transport Layer Security software when information is transmitted. Any hard copy data will be stored securely in lockable filing cabinets at the Federation Community Psychology Services at Federation University.

Eligible participants will be given access to a self-help dMH treatment program (Life Flex) and have their symptoms of anxiety or depression evaluated at day 15 to assess whether they meet the criteria to have their treatment program augmented with therapist assistance. Participants will be considered as eligible to be *stepped up* to a therapist-assisted condition if they meet any of the following criteria: they have not read the introduction module or module 1 of the Life Flex program; have symptoms of anxiety or depression that have deteriorated by >5 points (as assessed by comparison of their baseline depression [PHQ-9] and anxiety [GAD-7] scores against their week-3 scores); show symptom improvement, but their scores remain in a severe range; and are yet to complete the week-3 questionnaire assessment.

**Figure 1:**
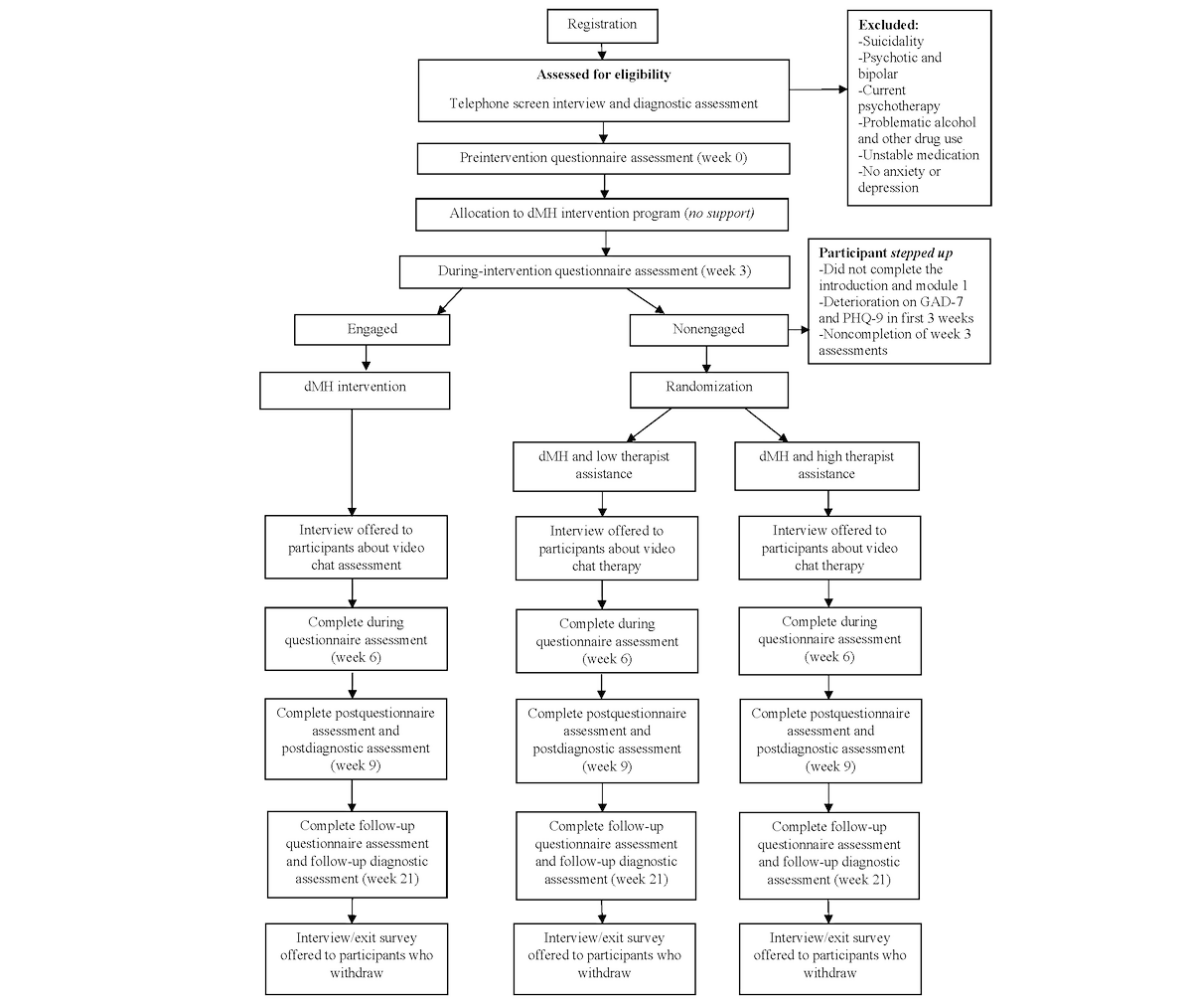
Study design diagram. dMH: digital mental health; GAD-7: Generalized Anxiety Disorder–7; PHQ-9: Patient Health Questionnaire–9.

Outcomes will be assessed before the intervention (week 0), during the intervention (weeks 3 and 6), after the intervention (week 9), and at the 3-month follow-up (week 21). Results will be disseminated via publication. Figure 1 illustrates the study’s design and expected participant flowchart.

### Participant Recruitment

#### Eligibility

Participants will be recruited from the Australian community and will be eligible for the trial if they meet the following criteria: aged ≥18 years, are Australian residents, have access to the internet, able to register their interest via the web using an email address, able to read and write in English, and have symptoms or a diagnosis of anxiety or depression (as assessed by the Mini International Neuropsychiatric Interview [MINI] 7.0.2 [13]).

Participants will be excluded if they are currently receiving psychological treatment for their mental health, have moderate to severe levels of alcohol or substance use, have active psychosis or active suicidal intent or plan, have unstable bipolar disorder (as assessed by MINI 7.0.2 [13]), or take unstable doses of medication (ie, eligible participants will need to be on the same doses of their prescribed medications for at least one month before commencing the intervention).

#### Screening Procedures

Potential participants will register their interest to participate in the study via the completion of a screening contact form on the study’s website. Interested participants will be presented with the Plain Language Information Statement before completing the contact form. Consent will be obtained at 2 intervals (upon initial completion of the contact form and again when entered into the trial), whereby participants will be required to click a button, *I agree*, indicating that they consent to the study. After consent is obtained, participants will be invited to participate in a brief telephone screening interview that corresponds with the *Diagnostic and Statistical Manual for Mental Disorders, Fifth Edition* (DSM-5) [14] criteria for generalized anxiety disorder, social phobia, panic disorder, agoraphobia, and major depressive disorder. During the telephone interview, the participants’ ability to attend a video chat assessment will be determined. The interview will conclude with exploration of their motivation to improve their symptoms of anxiety or depression.

Eligible participants will be scheduled for a diagnostic assessment. That is, administration of MINI 7.0.2 [13] via video chat technology accessed through the My Digital Health platform of Federation University. This clinical assessment will determine the presence of mental health symptoms (allowing for assessment of the inclusion and exclusion criteria of the study) and assignment of clinical severity ratings for any identified disorders. MINI is a short, structured interviewing tool designed to aid DSM-5 diagnosis and monitor the clinical outcomes of the 17 most common mental disorders. It has a mean administration time of 15 minutes, with questions requiring a “yes” or “no” response (eg, “In the past two weeks, were you much less interested in most things or much less able to enjoy the things you used to enjoy, most of the time?”). MINI has been validated against the long Structured Clinical Interview for Diagnostic and Statistical Manual for Mental Disorders diagnoses and has been identified as a more time-efficient diagnostic tool, demonstrating excellent interrater reliability (*k*=0.88-1) [13]. MINI will be administered before the intervention (week 0); after the intervention (week 9); and 3 months after the treatment, at follow-up (week 21).

### Participant Measures

#### Overview

Eligible participants will be given access to the preintervention (week 0) self-report questionnaire assessment comprising a demographic questionnaire (including age, sex, relationship status, education level, alcohol and other drug use, medication use, and current and past access to mental health services). Participants will also be asked to complete a short questionnaire package at weeks 3 and 6 and outcome measures after the intervention (week 9) and at the 3-month follow-up (week 21). At week 3, participants’ questionnaire data for anxiety (GAD-7) and depression (PHQ-9) will be compared against the preintervention outcome data to assess whether the stepped care rules of the trial have been met and whether a therapist is required to be assigned to a participant. Table 1 shows the schedule and frequency of the assessments during the trial for participants.

**Table 1:** Schedule of enrollment and assessments.

Concepts and measures			Preintervention period (week 0)		Midintervention period (weeks 3 and 6)		Postintervention period (week 9)		Follow-up period (week 21)
**Screening**									
	Anxiety and depression screen (DSM-5^a^)	✓							
	Diagnosis (MINI^b^ 7.0.2)	✓				✓		✓	
**Primary outcomes**									
	Anxiety (GAD-7^c^)	✓		✓		✓		✓	
	Depression (PHQ-9^d^)	✓		✓		✓		✓	
**Secondary outcomes**									
	Working alliance (WAI-S^e^)			✓		✓			
	Health status (AQol^f^)	✓				✓		✓	
	Health resources			✓		✓			
**Moderators**									
	Motivation (CMOTS^g^)	✓				✓			
	Self-efficacy (BPSES^h^)	✓				✓			
	Demographic questionnaire	✓				✓		✓	
**Treatment**									
	System Usability Scale			✓					
	Preferences questionnaire	✓							
	Satisfaction questionnaire					✓			
	Semistructured feedback interviews			✓		✓			

^a^DSM-5: *Diagnostic and Statistical Manual for Mental Disorders, Fifth Edition*.

^b^MINI: Mini International Neuropsychiatric Interview.

^c^GAD-7: Generalized Anxiety Disorder–7.

^d^PHQ-9: Patient Health Questionnaire–9.

^e^WAI-S: Working Alliance Inventory–Short.

^f^AQol: Assessment of Quality of Life.

^g^CMOTS: Client Motivation for Therapy Scale.

^h^BPSES: Bipolar Self-efficacy Scale.

#### Anxiety

The GAD-7 [15] is a self-report measure that assesses anxiety symptoms over the past 2-week period. Total scores range from 0 to 21, with high scores suggestive of more severe anxiety symptoms. Each item is rated on a 4-point scale, with scores >8 indicative of clinically significant anxiety symptoms, warranting further evaluation. Scores of 5, 10, and 15 represent the cutoff points for mild, moderate, and severe anxiety symptoms, respectively. The GAD-7 is sensitive to changes in generalized anxiety disorder, social phobia, panic disorder, and posttraumatic stress disorder, with a score of >5 points indicative of clinically meaningful change over time [15]. The GAD-7 has strong psychometric properties [15].

#### Depression

The PHQ-9 [16] is a self-report measure designed to assess symptoms of major depressive disorder over the past 2-week period. Total scores range from 0 to 27, with high scores indicative of great depressive symptoms. Each item is rated on a 4-point scale, with scores >10 used as cutoff to indicate the presence of clinically significant depressive symptoms. Scores of 5, 10, 15, and 20 represent the cutoff points for mild, moderate, moderately severe, and severe depressive symptoms, respectively. The PHQ-9 has strong psychometric properties [16].

#### Working Alliance

The Working Alliance Inventory–Short (WAI-S) [17] is an abbreviated version of the Working Alliance Inventory based on the theory by Bordin [18] and comprises the following subscales: goal agreement, task agreement, and bond. In this study, the WAI-S is available in a therapist version (10-item scale) and a participant version (12-item scale), with both versions to be used to capture the strength of the therapeutic alliance from both perspectives. Total scores range from 12 to 60, with high scores suggestive of strong therapeutic alliance. Each item is rated on a 5-point scale, and the measure has strong psychometric properties [17]. To assess participants’ technological alliance with the self-help dMH program, the wording of the items of the WAI-S have been slightly modified for this study, to capture participant ratings for a dMH program without therapist assistance.

#### Health Status

The Assessment of Quality of Life (AQol) [19] is a 12-item, self-report measure designed to assess ratings of health-related quality of life in a variety of life areas such as independent living, illness, social relationships, psychological well-being, and physical senses. The AQol can be scored in 2 ways—as a psychometric measure and as a utility measure. As a psychometric measure, scores are used to derive a simple psychometric score for health-related quality of life and provide profile scores on each of the 5 dimensions. When the AQol is scored as a utility measure, it can provide dimension scores and an overall index of the health state, which can be used in economic evaluation and cost analysis. The AQol has been validated across several countries, has been shown to have high degree of internal consistency (Cronbach α=.81), and is deemed an acceptable measure of health-related quality of life.

#### Health Resources

A Health Care Resource Usage questionnaire for cost-effectiveness has been specifically designed for the study to evaluate participants’ primary and secondary health care consultations and admissions, use of antidepressant and antianxiolytic medication, and resource use associated with treatment received in the trial. The treatment received in the trial will encompass the amount of therapist time required, supervisor’s time, internet access, and computer use.

#### Motivation

The Client Motivation for Therapy Scale (CMOTS) [20] is a self-report measure designed to assess the degree to which an individual is motivated for therapy and the impact of a person’s motivation on treatment effectiveness and mental health symptoms. The 24 items assess 6 dimensions of the self-determination continuum of motivation proposed by Deci and Ryan [21]: intrinsic motivation, 4 forms of extrinsic motivation (integration, identification, introjection, and external regulation), and amotivation for therapy. The CMOTS obtains subscale scores for each type of motivation, with high scores reflecting high levels of each type of motivation. Each item is rated on a 7-point scale. The CMOTS has strong psychometric properties [20].

#### Self-efficacy

The Bipolar Self-efficacy Scale (BPSES)–Modified [22] has been slightly modified for use in this study to measure the self-efficacy of participants with anxiety and depressive symptoms opposed to bipolar disorder symptoms. The 17 items of the modified self-report measure of self-efficacy assess how confident an individual feels in performing a range of activities related to their mental health such as taking their medication as prescribed, maintaining regular hours of sleep, and identifying early warning signs of anxiety and depression (as opposed to bipolar symptoms). Each item is rated on a 10-point scale, with high scores indicative of high levels of self-efficacy. The BPSES has strong psychometric properties, with sensitivity to detect change over the course of treatment [22].

#### Usability

The System Usability Scale [23] is a 10-item measure designed to assess participant perceptions of the usability of a program. The System Usability Scale will be used in this study to assess participants’ ease and perceptions about the dMH program’s usability (eg, “I thought the program was easy to use”). Each item is rated on a 5-point scale, with high scores reflective of increased system usability.

#### Preferences

A bespoke preferences questionnaire has been purposively developed to assess participants’ preferences for treatment before commencing the trial. Participants are presented with brief information about each of the 3 treatment conditions (dMH program only, low intensity therapist assistance, and high intensity therapist assistance), which they are then asked to rank in order of their preference.

#### Satisfaction

A purposively designed 10-item satisfaction questionnaire has also been developed to assess participants’ level of satisfaction with the treatment received. Overall, 8 of the 10 items are rated on a 4-point scale. For the other 2 questions, participants are asked to qualitatively share what they believe were the best and worst parts of the dMH treatment program.

### Primary Outcomes

The primary outcomes will be participants’ level of anxiety (GAD-7 [15]) and depression (PHQ-9 [16]).

### Secondary Outcomes

The secondary outcomes being assessed are working alliance (WAI-S [17]), health status (AQol [19]), health resources, self-efficacy (BPSES [22]), and motivation (CMOTS [20]).

### Treatment Conditions

#### Life Flex Program

The primary dMH intervention in each of the 3 treatment conditions is Life Flex, which is one of the dMH programs housed and offered via the My Digital Health platform. Life Flex is a third-wave transdiagnostic biopsychosocial treatment approach that aims to increase psychological flexibility, through cognitive behavior therapy, emotion regulation, and positive psychology principles, for anxiety and depression. The Life Flex program is suitable for individuals with or those without a DSM-5 diagnosis of anxiety or depression.

Life Flex teaches participants biopsychosocial *life skills*. It comprises 6 core modules, along with an introductory module and a booster module. The introductory module provides a rationale for the program, discusses brain plasticity and change processes, includes information about anxiety and depression, and explores factors contributing to motivation for change. Each of the 6 core modules focuses on increasing flexibility within a particular domain, such as biological (eg, breathing, nutrition, and sleep), emotional (eg, increasing emotional awareness), thinking (eg, identifying and challenging unhelpful cognitions), behavioral (eg, increasing pleasurable activities), wellness (eg, loving kindness), and life flexibility (eg, consolidating the life skills learned and goal setting). The booster session focuses on consolidation and reinforcement of learning. Further information about the Life Flex program can be accessed via a forthcoming paper that has recently been accepted for publication in JMIR Formative Research [24].

Each module of the Life Flex program will take approximately 25 minutes to complete. In addition, to reinforce the module-based information, there are 20 to 30 minutes of offline activities each week. Offline activities include applying the concepts and techniques discussed in the modules (eg, self-monitoring depressive and anxiety symptoms), undertaking one of the biological and wellness flexibility intervention strategies, monitoring emotions and thoughts, and undertaking the gradual exposure or behavioral activation activity. Participants will also receive automated emails (eg, to remind them to log in or to complete the questionnaires) and will be asked several questions at the beginning of each module to help gauge their progress. Module features include text, graphics, audio, video, editable forms, interactive games (eg, brain training), and downloads. Modules can be accessed via web, mobile phones, or tablet devices.

#### Life Flex Program–Only Condition

The treatment in step 1 of the trial will comprise the *program-only* Life Flex treatment program, whereby participants complete the fully automated, self-help program without therapist assistance. If participants do not improve in the *program-only* condition within the first 3 weeks (ie, no change in anxiety or depression symptoms, symptoms remain in the severe range, or noncompletion of the preintervention or week 3 questionnaire) or do not engage with the program (ie, noncompletion of the introduction module or module 1), they will be randomized to augment their program with either *low* therapist assistance (ie, one 10-minute support session per week) or *high* therapist assistance (ie, one 50-minute therapy session per week), delivered via video chat technology by provisionally or generally registered psychologists. Participants who improve or engage with the Life Flex program (program only) within the first 3 weeks maintain their no therapist assistance program. Participants who remain in the *program-only* treatment condition will complete the Life Flex program on a scheduled release design, with a new module being released every week, with the exception of module 4 (participants are given 2 weeks to work through and practice module-4 content).

#### Life Flex Program and Low-Intensity Therapist Assistance

Participants who are *stepped up* to the low-intensity therapist-assisted treatment condition will complete the same dMH treatment program (Life Flex), with the addition of a 10-minute weekly video chat session with a therapist for support and progress check-in of program content. The role of the therapist in the low-intensity treatment condition is to support the participant’s engagement with the Life Flex program by specifically checking participant progress with modules, task reinforcement, providing clarification where required, encouraging reading and skill practice, and ensuring that participants are clear about their between-session module tasks assigned for completion.

#### Life Flex Program and High-Intensity Therapist Assistance

Participants who are *stepped up* to the high-intensity therapist-assisted treatment condition will complete the same dMH treatment program (Life Flex), with the addition of a 50-minute weekly video chat therapy session with a therapist for individualized tailoring of the program content. Participants in the high-intensity treatment condition will have the opportunity to receive therapist guidance, tailored program content, questionnaire feedback, and support for generalizing skills to daily life and to engage in problem-solving where required.

Therapist assistance in both low-intensity and high-intensity conditions will be offered to participants for a period of up to 7 sessions. To ensure treatment fidelity and standardized treatment, the therapists will be provided with a treatment manual, with random checks of their recorded video chat sessions (eg, 20% of the total) by independent raters to determine adherence to the study protocol and treatment fidelity.

### Assessors and Therapists

#### Therapist Training Program

Before commencement of the trial, provisionally and generally registered psychologists will be required to complete and obtain competency in a purposively developed 14-hour, 5-module web-based training program covering an introduction to dMH interventions (specifically video chat technology), diagnostic assessments (covering MINI, anxiety, depressive disorders, and suicidality), qualitative interviewing techniques, and transdiagnostic cognitive behavior therapy principles that underpin the Life Flex dMH treatment program. The training program comprises a 40-item multiple-choice competency assessment, for which therapists are required to obtain a minimum score of 80%. The training program also contains a pre-evaluation and postevaluation survey designed to assess therapists’ knowledge about and confidence in the use of dMH interventions before and upon completion of the program and to obtain their satisfaction ratings of the training program.

#### Outcome Assessors and Trial Therapists

The assessors who complete the diagnostic clinical assessments and the therapists who deliver the low-intensity and high-intensity treatment assistance will be provisionally and generally registered psychologists who are on clinical placement at the Federation Community Psychology Services at Federation University in Ballarat, Victoria, Australia. A new assessor will be assigned for all 3 assessment time points and will differ from any therapist assigned to participants in both the low-intensity and high-intensity therapist-assisted conditions. The assessors will complete and be required to demonstrate competency in diagnostic assessments before administration, and both assessors and therapists will receive training and supervision from an experienced clinical psychologist throughout the trial.

Both assessment and therapist sessions will be audio or video recorded (with participants’ permission), so that random checking (eg, 20% of the total) can be completed to ensure adherence to administration, to enable agreement and interrater reliability of diagnoses, and to ensure that adherence to the therapist’s role (low or high intensity) is maintained. Adapted versions of the Internet-Delivered Cognitive Behavior Therapy–Therapist Rating Scale [25] and Cognitive Therapy Rating Scale (Multimedia Appendix 2) will be used by supervisors in the review process to ensure treatment fidelity in both treatment conditions. Each therapist will also be required to undertake regular supervision, where any remedial strategies to improve fidelity will be implemented. Remedial strategies will include the provision of constructive feedback, review of the components of treatment in sequence, further practice of skills where drift may have occurred, additional training (if necessary), review of initial fidelity training materials, and extra coaching or supervision.

#### Participant Interviews

Within the study, there will be 4 different types of interviews offered to participants depending on what is relevant to them: exploration of experiences of assessment via video chat technology, exploration of experiences of therapist assistance delivered via video chat technology, exploration of reasons for nonengagement with therapist assistance, and exploration of reasons for disengagement and withdrawal. Participants will be offered an interview at a suitable time and via their preferred mode: instant message, telephone, video chat, or email. Participants will also have the option to complete a brief exit survey instead of an interview if they withdraw from the study or choose not to engage with the offer of therapist assistance.

Participants who complete a diagnostic assessment or receive at least two therapist-assisted sessions via video chat will be invited to participate in a semistructured interview to reflect upon and share their experiences with the video chat interaction. They will be asked a series of questions focusing on their ability to develop a therapeutic alliance with their assessor and therapist, their views on video chat technology, exploration of any technological issues experienced, and overall strengths and limitations of video chat. The interviews will be conducted by the first author, focusing on asking questions in an open, curious, and reflective manner. If the interview is completed via telephone or video chat, it will be audio recorded (with participants’ permission) for qualitative analysis.

#### Assessor and Therapist Interviews

The provisionally and generally registered psychologists of the study will also be invited for interview to share their experiences with administering diagnostic assessments and delivering treatment via video chat technology and explore their training experiences. Assessors and therapists will be offered an interview at a suitable time and via their preferred mode: instant message, telephone, video chat, or email. They will be asked a series of questions focusing on their ability to develop a therapeutic alliance with their participants; their views about video chat technology; exploration of any technological issues experienced; exploration of implementation barriers and facilitators; and overall reflections about how a service delivered via video chat compares with an in-person, face-to-face service. The interviews will be conducted by the first author, and if completed via telephone or video chat, the interview will be audio recorded (with permission) for qualitative analysis.

### Randomization

The process of randomly allocating participants to either the low-intensity or high-intensity treatment groups will comply with CONSORT guidelines. A block randomization design will be used to randomly allocate each participant to either the low-intensity or high-intensity therapist assistance condition throughout the trial. Random allocation will occur when a participant has met the criteria to be *stepped up* to have their Life Flex program augmented with therapist assistance. Participants will be aware of the group they have been randomly allocated to but will remain unaware of the study’s objectives. The provisional or general psychologists will not be blinded to the treatment conditions, as they will need to adhere to either the support or the therapist role.

### Sample Size Calculation

Only aggregate data will be used for the main analysis. Assuming a conservative medium effect (ie, GPower f[v] test=0.25), significance set at 5% (*P*=.05), and power at 80%, 98 cases are required to demonstrate statistical significance in the primary outcome measures. However, allowing for a 30% attrition rate [26], a total target sample size of 137 participants will be required.

### Ethics Approval

The trial will be conducted in accordance with the ethical guidelines in the National Statement on Ethical Conduct in Human Research (National Statement on Human Research 2007; updated in 2018). Ethics approval for this project has been granted by the Federation University Australia Human Research Ethics Committee (A19-095), and this clinical trial is registered with the Australian and New Zealand Clinical Trials Registry (12620000422921).

## Results

Recruitment began in November 2020 and was completed at the end of March 2022. In total, 240 individuals registered for the clinical trial, of which 113 (47.1%) were assessed as eligible to commence the dMH intervention. At week 3, of the 113 participants, 73 (64.6%) met the stepped care criteria to have the dMH intervention augmented with therapist assistance, whereas 40 (35.4%) remained in the program-only condition. The results of the clinical trial are forthcoming as they have recently been accepted for publication in JMIR Publications [27].

Data will be entered, screened, and analyzed using statistical software. Intention-to-treat analysis will be used for primary outcomes, controlling for baseline differences where appropriate. The primary analysis will be conducted using 2 planned contrasts comparing a change in anxiety (GAD-7) and depression (PHQ-9) scores from preintervention assessment with those of the postintervention (week 9) and follow-up (week 21) assessments, using a mixed model repeated-measure analysis. A mixed model repeated-measure analysis is preferred owing to its ability to include participants with missing data. The study will also investigate whether reliable and clinically significant change has been achieved for participants following the recommendations of Jacobson and Traux [28]. Moderation analyses will be used to determine the role of self-efficacy and motivation in engagement and adherence and their effects on anxiety and depression outcomes in the 3 treatment conditions, and a *t* test will be used to determine the impact of preferences and treatment allocations on attrition rates.

Data obtained from open-ended questions about intervention satisfaction questionnaires and from interview questions will be transcribed verbatim from audio recordings into text and then analyzed using protocols of thematic analysis to identify common themes. Text transcripts will be read several times to identify emerging themes. Thematic analysis allows for the identification of themes that are driven by the data, using an experiential, bottom-up approach [29]. Close analysis of the data will suggest names for the themes, which are likely to represent something of meaning to participants and therapists, with direct quotes used to further illustrate meaning. Researchers will meet throughout the analysis process to review and discuss the data.

## Discussion

### Principal Findings

This study protocol presents an outline of the first adaptive intervention trial designed to determine the efficacy of various support intensities (ie, none, low, and high) of a dMH treatment program for anxiety and depression. The primary objective is to compare the efficacy of clinical outcomes (anxiety and depression) among the 3 treatment conditions. Specifically, it is predicted that therapist assistance delivered via video chat will be more effective in reducing anxiety and depression symptoms compared with program-only support. If this hypothesis is supported by the study findings, it will demonstrate that augmenting a dMH intervention with therapist assistance offered via video chat is beneficial for enhancing mental health outcomes within a community mental health treatment setting. The difference between low and high therapist assistance will also be evaluated. If the results indicate that the outcomes of these treatment conditions are similar, support will be provided for the implementation of low-intensity dMH interventions within the community, thus increasing timely community access to evidence-based interventions.

The study also has several secondary objectives that will be assessed. Examination of the role of motivation; self-efficacy; and participant preferences in engagement, adherence, and clinical outcomes among the 3 treatment conditions will be beneficial in improving our understanding of factors affecting participant attrition and engagement within dMH interventions. Qualitative evaluation of participant and therapist experiences and satisfaction with video chat technology is expected to provide further knowledge about the use of video chat for the assessment and treatment of anxiety and depression, specifically, the development of therapeutic relationships, implementation barriers and facilitators, and strengths and limitations of video chat technology. Furthermore, qualitative exploration of therapist training and implementation experiences in the delivery of dMH interventions is likely to improve the future adoption of dMH interventions incorporating video chat technology.

The findings of the trial have the potential to contribute toward the evidence base of adaptive treatment designs for anxiety and depression, with considerable implications for the implementation of stepped care models in dMH. As the study will evaluate outcomes of health resources and health status among the 3 treatment conditions, it is anticipated that the study results will enhance our understanding of participant needs and resources required at the self-help (dMH program only; step 1) and therapist-assisted (low and high therapist assistance; step 2) levels in the context of an adaptive intervention design.

### Limitations

Although the current trial is believed to contribute significantly to advancing the field of dMH interventions, the study is likely to have some limitations. Given the widely known high attrition rates in dMH treatment and the difficulties that people with depression and anxiety can face, such as avoidance and lack of motivation, participant recruitment and overall engagement may present a challenge. The study protocol has been designed with these considerations in mind, with the overall aim of reducing the impact of these challenges and furthering our knowledge about various support intensities of dMH treatment for anxiety and depression.

### Conclusions

This protocol outlines the first adaptive mixed methods clinical trial design investigating the efficacy of a 2-stage adaptive dMH intervention program incorporating both low-intensity and high-intensity therapist assistance delivered via video chat technology, compared with program-only support. This trial adds to the existing studies on dMH interventions and provides further understanding of participant and therapist experiences and satisfaction with video chat assessment and therapist assistance, along with therapist training and implementation experiences. Outcomes are expected to have implications for stepped care models and wide dissemination of dMH interventions for anxiety and depression.

## Appendix

Multimedia Appendix 1 Completed SPIRIT (Standard Protocol Items: Recommendations for Interventional Trials) checklist.

Multimedia Appendix 2 Cognitive Therapy Rating Scale manual.

## Abbreviations

AQol: Assessment of Quality of Life
BPSES: Bipolar Self-efficacy Scale
CMOTS: Client Motivation for Therapy Scale
CONSORT: Consolidated Standards of Reporting Trials
dMH: digital mental health
DSM-5: *Diagnostic and Statistical Manual for Mental Disorders, Fifth Edition*
GAD-7: Generalized Anxiety Disorder–7
MINI: Mini International Neuropsychiatric Interview
PHQ-9: Patient Health Questionnaire–9
SPIRIT: Standard Protocol Items: Recommendations for Interventional Trials
WAI-S: Working Alliance Inventory–Short

## Data Availability

Deidentified aggregate data sets generated or analyzed during this trial may be made available from the corresponding author upon reasonable request and if data sharing is in accordance with the study’s ethics approval and general data protection regulations.
