# Supplementary material for: Evaluation of Various Support Intensities of Digital Mental Health Treatment for Reducing Anxiety and Depression in Adults: Protocol for a Mixed Methods, Adaptive, Randomized Clinical Trial
Source: JMIR Res Protoc. 2023 Apr 28;12:e45040. doi: 10.2196/45040 (PMC10182448; doi:10.2196/45040)
Supplement: Multimedia Appendix 2 [file resprot_v12i1e45040_app2.pdf]

COGNITIVE THERAPY SCALE

RATING MANUAL

Jeffrey Young, Ph.D.

Aaron T. Beck, M.D.

Revised Draft

©1980 by J. E. Young & A. T. Beck

Beck Institute for Cognitive Behavior Therapy
One Belmont Avenue, Suite 700 | Bala Cynwyd, PA 19004 | 610-664-3020
beckinstitute.org

GENERAL INSTRUCTIONS TO RATERS

1. The most serious problem we have observed in raters is a "*halo effect*". When the rater thinks the therapist is good, he/she tends to rate the therapist high on all categories. The reverse is true when the rater believes the session is bad.

   One of the most important functions of the Cognitive Therapy Scale is to identify the therapist's specific strengths and weaknesses. It is rare to find a therapist who is uniformly good or bad. It may be helpful, therefore, for raters to list positive and negative observations as they listen to a session, rather than concentrate on forming one global impression.

2. A second problem is the tendency of some raters to rely solely on their own notions of what a particular scale point means (e.g., 4 is average) and to disregard the descriptions provided on the form. The problem with this is that we each attach idiosyncratic meanings to particular numbers on the 6-point scale. The most critical raters assign a 1 whenever the therapist is "unsatisfactory", while the most generous raters assign a 5 when the therapist has merely "done a good job" or "tried hard".

   The descriptions on the scale should help to insure more uniformity across raters. Therefore, we urge you to base your numerical ratings on the descriptions provided whenever possible. Do not be concerned if the resulting numerical score does not match your overall "gut feeling" about the therapist. (After all, you are free to express your "gut feeling" in the overall rating on the first page.)

   The only exception should be in sessions where the descriptions do not seem to describe the specific therapist problems and behaviors you observed. When this is the case, disregard the specific descriptions and rely on the more general scale descriptions supplied in the directions. With these exceptions, it would be helpful if raters noted why the descriptions did not seem to apply, so the scale can be refined in the future.

1. AGENDA

Objective:

Because cognitive therapy is a relatively short-term, problem-solving therapy, the limited time available for each interview must be used judiciously. At the beginning of each session, the therapist and patient together establish an agenda with specific target problems to focus on during each session. The agenda helps insure that the most pertinent issues are addressed in an efficient manner.

Background Material:

Cognitive Therapy of Depression, pp. 77-78, 93-98, 167-208; *Cognitive Therapy and the Emotional Disorders,* pp. 224-300.

Desirable Therapist Strategies:

The agenda usually begins with a brief resume of the patient's experiences since last session. This resume includes relevant events of the past week, discussion and feedback regarding homework, and the patient's current emotional status (as indicated by the BDI score, Anxiety Checklist score, and patient's verbal report of progress).

Because cognitive therapy is relatively short-term, it relies heavily on the pinpointing of specific target problems. Without target problems, therapy is much less focused, much less efficient, and therefore much slower. If the target problem is not chosen properly, the therapist may find it very difficult to make headway, either because a more central problem is interfering with progress or because the patient is not sufficiently concerned about the problem to cooperate fully. In some cases, a target problem may be central, yet not be amenable to treatment at a given point in therapy.

At the beginning of a session, therefore, the patient and therapist together develop a list of problems that they would like to work on during the hour. These might include specific depressive symptoms, such as apathy and lack of motivation, crying, or difficulty concentrating; to external problems in the patient's life, such as marital problems, career issues, child-rearing concerns, or financial difficulties.

After the list of possible topics has been completed, the patient and therapist discuss and reach conclusions about which topics to include, the order to cover them, and, if necessary, how much time should be allotted to each topic. Some of the considerations in setting priorities are: the stage of therapy, the severity of the depression, the presence of suicidal wishes, the degree of distress associated with each problem area, the likelihood of making progress in solving, the problem, and the number of different life areas affected by a particular theme or topic.

Some of the most common mistakes we observe in novice cognitive therapists are:

1) failure to agree on specific problems to focus on;

2) selection of a peripheral problem to attack rather than a central concern;

3) a tendency to skip from problem to problem across sessions rather than persistently seek a satisfactory solution to one or two problems at a time.

Generally, in the earlier phases of treatment and in working with more severely depressed patients, behavioral goals are likely to be more useful than strictly cognitive ones. As therapy progresses, the emphasis often switches from relieving specific depressive symptoms (such as inactivity, excessive self-criticism, hopelessness, crying, and difficulty concentrating) to broader problems (such as anxiety about work, life goals, and interpersonal conflicts).

The process of selecting a target problem usually involves a certain degree of "trial and error." The therapist should attempt to follow the agenda throughout the session. However, the therapist and patient should be willing to switch to a different problem occasionally if it becomes apparent that the one they have selected is less important or not yet amenable to change. However, a switch in target problem should be a collaborative decision and should follow a discussion of the rationale for changing topics. If the therapist switches without explanation, it may be perceived by the patient as evidence that the problem cannot be solved and is hopeless.

The therapist must also be sensitive to patients' occasional desires to discuss or "ventilate" regarding issues that are important to the patient at the particular moment, even though such discussions may not seem to offer much relief in the long run or may seem irrelevant to the therapist. Such flexibility epitomizes the collaborative relationship in cognitive therapy.

Agenda-setting should be done quickly and efficiently. The therapist should avoid discussing the content of specific agenda items with the patient prior to completing the agenda. Furthermore, the agenda should not be overly ambitious; it is usually impossible to cover more than one or two target problems in a given session. When done properly, the agenda can usually be set within five minutes.

2. FEEDBACK

Objective:

The therapist should work to carefully elicit the patient's positive and negative reactions to all aspects of therapy. Feedback also includes checking to be sure that the patient understands the therapist's interventions, formulations and line of reasoning, and the therapist has accurately understood the patient's main points.

Background Material:

Cognitive Therapy of Depression, pp. 81-84.

Desirable Therapist Strategies:

The cognitive therapist strives throughout each session to be certain that the patient is responding positively to the therapeutic process. Beginning with the first session, the therapist carefully elicits the patient's thoughts and feelings about all aspects of therapy. He/she routinely asks for the patient's **evaluation of each session**, and encourages the patient to express any **negative reactions to the therapist**, to the way a particular problem is handled, to homework assignments, etc. The therapist must also be sensitive to negative covert reactions to the interviews expressed verbally or nonverbally by the patient, and should ask for the patient's thoughts when such clues are noticed. Whenever possible, the therapist should ask the patient for suggestions about how to proceed, or to choose among alternative courses of action.

A final feature of the feedback process is for the therapist to **check continually to be certain that the patient understands the therapist's formulations**. Depressed patients often indicate understanding simply out of compliance. Thus, the therapist should regularly provide **capsule summaries** of what has happened during the session and ask the patient to abstract the main points from the therapy session. In fact, it is often helpful to have the patient write down these conclusions to review during the week. Similarly, it is important for the therapist to summarize regularly what he/she believes the patient is saying and to ask the patient to modify, correct, or "fine tune" the therapist's summary.

3. UNDERSTANDING

Objective:

The therapist accurately communicates an understanding of the patient's thoughts and feelings. "Understanding" refers to how well the therapist can step into the patient's world, see and experience life the way the patient does, and convey this understanding to the patient. Understanding incorporates what other authors have referred to as listening and empathic skills.

Background Material:

Cognitive Therapy of Depression, pp. 47-49.

Rationale:

The ineffective therapist often misinterprets or ignores the patient's view and incorrectly projects his/her own attitudes, conventional attitudes, or attitudes derived from a particular theoretical system onto the patient. When this happens, the interventions will probably fail since they will be directed at cognitions or behaviors that are not really central to the patient's view of reality.

Desirable Therapist Strategies:

The therapist should be sensitive both to what the patient explicitly says and to what the patient conveys through tone of voice and non-verbal responses. Sometimes, for example, a patient may not recognize or verbalize a particular feeling (such as anger) and yet may communicate the emotion to the therapist through his/her tone of voice in describing a particular event or person.

Unless the therapist is able to grasp the patient's "internal reality", it is unlikely that he/she will be able to intervene effectively. Furthermore, it will be difficult for the therapist to establish rapport unless the patient believes that the therapist understands him/her. The therapist can convey this understanding by rephrasing or summarizing what the patient seems to be feeling. The therapist's tone of voice and non-verbal responses should convey a sympathetic understanding of the patient's point of view (although the therapist must maintain objectivity toward the patient's problems).

Ideally, the therapist's understanding of the patient's "internal reality" will lead to an accurate conceptualization of the patient's problems and then to an effective strategy for change.

Special Considerations in Rating:

"Understanding" seems to be one of the most difficult categories in terms of achieving interrater agreement. It is important, therefore, that raters pay special attention to the descriptions for each scale point. The 0 level means that the therapist **completely missed the point** of what the patient was saying. To score "0" the therapist fails to repeat accurately even the most obvious elements of what the patient says. The 2 level applies to therapists who are too literate or tangential -- they are able to reflect what the patient explicitly says, but either seem dense regarding more subtle connotations that suggest something else is going on or they accurately repeat peripheral aspects of what the patient says but they miss the main point.

The 4 and 6 levels both indicate that the therapist seems to grasp the patient's perspective. The 6 level, however, indicates both greater skill at communicating a sympathetic understanding to the patient and a keener grasp of the patient's world that may be reflected in the therapist's ability to predict how and why the patient reacts as he/she does in particular situations.

4. INTERPERSONAL EFFECTIVENESS

Objective:

The cognitive therapist should display optimal levels of warmth, concern, confidence genuineness, and professionalism.

Background Material:

Cognitive Therapy of Depression. pp. 45-47, 49-50.

Rationale:

A variety of research studies support the importance of these "non-specific" variables in favorable outcomes of psychotherapy. For cognitive therapists, these interpersonal skills are essential in establishing collaboration.

Desirable Therapist Strategies:

The cognitive therapist should be able to communicate that he/she is **genuine**, sincere, and open. The therapist should not act in a manner that seems patronizing or condescending, not should he/she evade patients' questions. Thus, the experienced cognitive therapist does not seem to be playing the role of a therapist, but comes across as straightforward and direct.

Coupled with this openness, cognitive therapists should convey warmth and concern through the content of what they say and through such non-verbal behaviors as tone of voice and eye contact. Therapists must be careful that, in the course of questioning the patient's point of view they do not seem to be critical of, disapproving of, or ridiculing the patient's perspective. The therapist can often use and encourage humor in establishing a positive relationship.

It is also vital for therapists to display a professional manner. Without seeming distant or cold, the cognitive therapist must convey a relaxed confidence about his/her ability to help the depressed patient. This confidence can serve as a partial antidote to the patient's initial hopelessness about the fixture. A professional manner may also make it easier for the therapist to take a directive role, impose structure, and be convincing in expressing alternative points of view. Although the patient and therapist share responsibility for the therapy, the effective therapist must be able to use the leverage accorded him as the professional when necessary.

Special Considerations in Rating:

Interpersonal effectiveness is another category in which interrater agreement has been less than ideal. The 0 level should be used for therapists who could reasonably be expected to have negative effects on the patient because of their poor interpersonal skills. Such therapists, because they are hostile, cold, or critical, may undermine the patient's self-esteem and make the development of trust impossible. The 2 level is intended for therapists who are not likely to be destructive to the patient, but who may hinder therapy progress by being impatient, insincere, aloof, or by not seeming competent. Such therapists will not be able to use the leverage available to therapists who are able to build a stronger relationship with their patients. 4 and 6 levels both represent interpersonal skills; the difference is simply one of degree.

5. COLLABORATION

Objective:

One of the fundamental precepts of cognitive therapy is that there be a collaborative relationship between the patient and therapist. This collaboration takes the form of a therapeutic alliance in which the therapist and patient work together to fight a common enemy: the patient's distress.

Background Material:

Cognitive Therapy and the Emotional Disorders, pp. 220-221; *Cognitive Therapy of Depression,* pp. 50-54.

Rationale:

There are at least three goals of this collaborative approach. First, collaboration helps insure that the patient and therapist have compatible goals at each point in the course of treatment. Thus, they will not be working at cross purposes. Second, the process minimizes patient resistance that often arises when the therapist is viewed as a competitor or an aggressor, or is seen as trying to control or dominate the patient. Third, the alliance helps prevent misunderstandings between the patient and therapist. Such misunderstandings can lead the therapist to go down blind alleys or can lead the patient to misinterpret what the therapist has been trying to convey.

Desirable Therapist Strategies:

Rapport: Rapport refers to harmonious accord between people. In cognitive therapy, this rapport involves a sense that the patient and therapist are functioning together as a team, that they are comfortable working together. Neither is defensive or unduly inhibited. To develop rapport, the therapist will often need to exhibit the understanding and interpersonal qualities described in items 2, 3, and 4 on the Cognitive Therapy Scale. Rapport, however, involves more than showing warmth and empathy. It requires that the therapist adapt the structure and style of the therapy to the needs and desires of each particular patient.

Balancing structure against patient autonomy: To establish a collaborative relationship, the therapist needs to strike a balance between being directive and imposing structure on the one hand, and allowing the patient to make choices and take responsibility on the other. This balance involves deciding when to talk and when to listen; when to confront and when to back off; when to offer suggestions and when to wait for the patient to make his/her own suggestions.

Focusing on problems both patient and therapist consider important: One of the most important aspects of collaboration is the knowledge that the session is focused on a problem that both patient and therapist consider important. Unless the therapist is attentive to the patient's desires in each session, he/she may persist in focusing on a problem or technique that the patient does not consider relevant or important. The patient and therapist may begin to work at cross purposes and the collaboration can break down.

Explaining the rationale for interventions: Another element of the collaborative process is for the therapist to explain the rationale for most interventions he/she makes. This rationale demystifies the process of therapy and thus makes it easier for the patient to understand a particular approach. Furthermore, when the patient can see the relationship between a particular homework assignment or technique and the solution to his/her problem, it is more likely that the patient will participate conscientiously.

6. PACING AND EFFICIENT USE OF TIME

Objective:

The therapist should accomplish as much as possible during each session, taking into account the present capacity of the patient to absorb new information. To optimize the available time, the therapist must maintain sufficient control, limit discussion of peripheral issues, interrupt unproductive discussions, and pace the session appropriately.

Background Material:

Cognitive Therapy of Depression, pp. 65-66.

Desirable Therapist Strategies:

We have often observed sessions in which the therapist paced the session much too slowly or too rapidly for a particular patient. On the other hand, the therapist may belabor a point after the patient has already grasped the message or may gather much more data than is necessary before formulating a strategy for change. In these cases, the sessions seem painfully slow and inefficient. On the other hand, the therapist may switch from topic to topic too rapidly, before the patient has had an opportunity to integrate a new perspective. Or the therapist may intervene before he/she has gathered enough data to conceptualize the problem.

The agenda provides a structural plan that should help the therapist use time efficiently. The therapist should monitor the flow of discussion and **maintain sufficient control** over the process of each session to ensure that both patient and therapist adhere to their original plan. In so doing, the most important agenda items will be covered. Unfinished business should be rescheduled.

During agenda-setting, the therapist's input can **limit discussion of peripheral issues**. However, during the session, the patient and therapist may inadvertently drift from the critical agenda topic to a related, yet less important item. In such cases, the therapist should politely interrupt these peripheral discussions and return to the agenda item.

Even when focused on a central issue, the therapy discussion may reach a point when progress is no longer being made. In such cases, the therapist should gently interrupt the unproductive discussion and try to approach the issue from another perspective.

7. GUIDED DISCOVERY

Objective:

Guided discovery is one of the most basic strategies of the effective cognitive therapist. The cognitive therapist often uses exploration and questioning to help patients see new perspectives where other therapists use debating or lecturing. The cognitive therapist attempts to avoid "cross-examining" the patient or putting the patient on the defensive.

Background Material:

Cognitive Therapy of Depression, pp. 66-71.

Rationale:

We have observed that patients often adopt new perspectives more readily when they come to their own conclusions than when the therapist tries to debate with the patient. In this respect, the cognitive therapist is more like a skilled teacher than a lawyer. He/she guides the "student" to see logical problems in the student's present position; to examine evidence that contradicts the students beliefs; to gather information when more is necessary to test a hypothesis; to look at new alternatives that the student may never have considered, and to reach valid conclusions after this exploration. The techniques for changing cognitions and behaviors in this therapy can for the most part be subsumed within this more basic strategy, which educators label "guided discovery". Thus, hypothesis testing, empiricism, setting up experiments, inductive questioning, weighing advantages and disadvantages, etc., are all tools at the therapist's disposal to aid in the process of "guided discovery."

Desirable Therapist Strategies:

Questioning deserves special attention since it is so critical to the process of guided discovery. Skillfully-phrased questions presented in a logical sequence are often extremely effective. A single question can simultaneously make the patient aware of a particular problem area, help the therapist evaluate the patient's reaction to this new area of inquiry, obtain specific data about the problem, generate possible solutions to problems that the patient had viewed as insoluble, and cast serious doubt in the patient's mind regarding previously distorted conclusions.

Some of the functions that questioning may serve in this process are outlined below:

1. To encourage the patient to begin the decision-making process by developing alternative approaches.

2. To assist the patient in resolving a decision by weighing the pros and cons of alternatives that have already been generated, thus narrowing the range of desirable possibilities.
3. To prompt the patient to consider the consequences of continuing to engage in dysfunctional behaviors.
4. To examine the potential advantages to behaving in more adaptive ways.
5. To determine the **meaning** the patient attaches to a particular event or set of circumstances.
6. To help the patient define criteria for applying certain maladaptive self-appraisals (see the discussion of the technique of operationalizing a negative construct in Section 9).
7. To demonstrate to the patient how he/she is selectively focusing on only negative information in drawing conclusions. In the excerpt that follows, a depressed patient was disgusted with herself for eating candy when she was on a diet.

    Patient: I don't have any self-control at all.

    Therapist: On what basis do you say that?

    Patient: Somebody offered me candy and I couldn't refuse it.

    Therapist: Were you eating candy every day?

    Patient: No, I ate it just this once.

    Therapist: Did you do anything constructive during the past week to adhere to your diet?

    Patient: Well, I didn't give in to the temptation to buy candy every time I saw it at the store...Also, I did not eat any candy except the one time it was offered to me and I felt I couldn't refuse it.

    Therapist: If you counted up the number of times you controlled yourself versus the number of times you gave in, what ratio would you get?

    Patient: About 100 to 1.

    Therapist: So if you controlled yourself 100 times and did not control yourself just once, would that be a sign that you are weak through and through?

    Patient: I guess not -- not through and through (smiles).

8. To illustrate to the patient the way in which he/she disqualifies positive evidence. In the example below, the patient recognizes that he has ignored clear-cut evidence of improvement.

    Patient: I really haven't made any progress in therapy.

    Therapist: Didn't you have to improve in order to leave the hospital and go back to college?

    Patient: What's the big deal about going to college every day?

    Therapist: Why do you say that?

    Patient: It's easy to attend these classes because all the people are healthy.

    Therapist: How about when you were in group therapy in the hospital? What did you feel then?

    Patient: I guess I thought then that it was easy to be with the other people because they were all as crazy as I was.

    Therapist: Is it possible that whatever you accomplish you tend to discredit?

9. To open for discussion certain problem areas that the patient had prematurely reached closure on, and which continue to influence his/her maladaptive patterns.

This is not to say that the effective cognitive therapist relies solely, or even primarily, on questioning in all sessions. In some instances, it is appropriate for the therapist to provide information, confront, explain, self-disclose, etc. rather than question. The balance between questioning and other modes of intervention on the particular problem being dealt with, the particular patient, and the point in therapy. The appropriateness of an intervention can be assessed by observing: its effect on the collaborative relationship; the degree of dependency it promotes on the patient; and, of course, its success in helping the patient adopt a new perspective.

There is often a fine line between **guiding** a patient and trying to **persuade** a patient. In some instances, the cognitive therapist may need to reiterate forcefully a point that the therapist and patient have already established. The main distinction, then, in deciding whether a therapist is acting in a desirable manner is not whether the therapist is forceful or tenacious but whether the therapist overall seems to be **collaborating** with the patient rather than **arguing** with the patient. In the excerpt that follows, the therapist uses questioning to demonstrate to the patient the maladaptive consequences of holding the assumption that one should **always** work up to one's potential.

Patient: I guess I believe that I should always work up to my potential.

Therapist: Why is that?

Patient: Otherwise I'd be wasting time.

Therapist: But what is the long-range goal in working up to your potential?

Patient: (*Long pause.*) I've never really thought about that. I've just always assumed that I should.

Therapist: Are there any positive things you give up by always having to work up to your potential?

Patient: I suppose it make it hard to relax or take a vacation.

Therapist: What about "living up to your potential" to enjoy yourself and relax? Is that important at all?

Patient: I've never really thought of it that way.

Therapist: Maybe we can work on giving yourself permission not to work up to your potential at all times.

Example of an Undesirable Application:

The desirable applications above can be contrasted with one of the most common stylistic errors we observe in trainees. The therapist's behavior sometimes inappropriately resembles that of a high pressure salesman, persuading patients that they should adopt the therapist's point of view. For contrast, here is a brief example of the "high pressure" approach:

Patient: I just can't do anything right in school anymore.

Therapist: That's easy to understand. You're depressed. And when people are depressed, they have a hard time studying.

Patient: I think I'm just stupid.

Therapist: But you did very well up until a year ago, when your father died and you got depressed.

Patient: That's because the work was easier then.

Therapist: Surely there must be something you are doing right in school. You're probably exaggerating.

8. FOCUSING ON KEY COGNITIONS AND BEHAVIORS

Objective and Rationale:

Once the therapist and patient have agreed on a central target problem, the next step is for the therapist to conceptualize why the patient is having difficulty in this particular area. In order to conceptualize this problem, the therapist must elicit and identify the key automatic thoughts, underlying assumptions, behaviors, etc. that comprise the problem. These specific cognitions and behaviors then serve as targets for intervention.

Background Material:

Cognitive Therapy and the Emotional Disorders pp. 6-131, 246-257; *Cognitive Therapy of Depression pp.* 142-152, 163-166, 244-252.

Conceptualizing the Problem:

The effective cognitive therapist is continually engaged in the process of **conceptualizing the patient's problem** while he/she is helping the patient identify key automatic thoughts, assumptions, behaviors, etc. Through this conceptualization, the therapist integrates specific cognitions, emotions, and behaviors into a broader framework that explains why the patient is having difficulty in a particular problem area. Without this broader framework (which may undergo continued revision) the therapist is like a detective who has a lot of clues but still has not solved the mystery. (Once the clues are pieced together, though, the nature of the "crime" becomes clear.) The therapist can then distinguish between thoughts and behaviors that are central to the probing and those that are peripheral. The conceptualization therefore guides the therapist in deciding which automatic thoughts, assumptions, or behaviors to focus on first, and which to postpone until a later date. Without such conceptualization, the therapist may select cognitions or behaviors in a "hit-or-miss" fashion and therefore make limited or erratic progress.

Although the quality of a therapist's conceptualizing is difficult to assess from observing a single session, we believe that in the long run it proves to be one of the most crucial determinants of the effectiveness of a cognitive therapist. **We try to make inferences about the quality of the conceptualization by observing whether the specific conditions or behaviors focused on in a given session seem to be central to the patient's problem rather than peripheral**. If the therapist's conceptualization is poor (we hypothesize), then the rationale for focusing on a particular thought or behavior will not be clear to the experienced rater. Furthermore, target problems, interventions, homework, etc. will appear to "hang together" in a unified framework if the conceptualization is good.

Desirable Therapist Strategies for Eliciting Automatic Thoughts

Inductive Questioning

The therapist can ask the patient a series of questions designed to explore some of the possible reasons for the patient's emotional reactions. Skillful questioning can provide patients with a strategy for introspective exploration that they can later employ by themselves when the therapist is not nearby. (See the example in the section on guided discovery).

Imagery

When patients can identify events or situations that seem to trigger the emotional response, the therapist can suggest that the patients picture the distressing situation in detail. If the image is realistic and clear to the patients they are often able to identify the automatic thoughts they were having at the time. The excerpt below illustrates this technique:

Patient: I can't go bowling. Every time I go in there, I want to run away.

Therapist: Do you remember any of the thoughts you had when you went there?

Patient: Not really. Maybe it just brings memories, I don't know.

Therapist: Let's try an experiment to see if we can discover what you were thinking. OK?

Patient: I guess so.

Therapist: I'd like you to relax and close your eyes. Now imagine you are entering the bowling alley. Describe for me what's happening.

Patient: (Describes entering the alley, getting a score sheet, etc.) I feel like I want to get out, just get away.

Therapist: What are you thinking now?

Patient: I'm thinking "Everyone I play with is going to laugh at me when they see how bad I play."

Therapist: Do you think that thought might have led to your wish to run away?

Patient: I know it did.

Role Playing:

When the trigger event is interpersonal in nature, role-playing is often more effective than imagery. With this strategy, the therapist plays the role of the other person involved in the upsetting situation, while patients "play" themselves. If patients can involve themselves in the role-play, the automatic thoughts can often be elicited with the assistance of the therapist.

Mood Shift During the Session:

The therapist can take advantage of any changes in mood that take place during the session by pointing them out to the patient as soon as possible. The therapist then asks the patient what he/she was thinking just prior to the increase in dysphoria, tears, anger, etc.

Daily Record of Dysfunctional Thoughts:

This is the simplest method of pinpointing automatic thoughts once the patient is familiar with the technique. The patient lists automatic thoughts at home in the appropriate column on the form. The therapist and patient review these thoughts during the session.

It is important to distinguish this process of eliciting automatic thoughts from the "interpretations" made in other psychotherapies. The cognitive therapist does not volunteer an automatic thought that the patient has not already mentioned. This "clairvoyance" undermines the patient's role as collaborator and makes it difficult for the patient to identify these thoughts at home when the therapist is not nearby. Even more important, if the therapist's "intuition" is wrong, he/she will be pursuing a blind alley. On occasion, it will be necessary for the therapist to suggest several plausible automatic thoughts (a multiple choice technique) when other strategies have failed.

The example of "clairvoyance" that follows provides a contrast to the imagery technique illustrated previously:

Patient: I can't go bowling. Every time I go in there, I want to run away.

Therapist: Why?

Patient: I don't know. I just want to leave.

Therapist: Do you tell yourself, "I wish I didn't have to bowl by myself'?

Patient: Maybe. I'm not sure.

Therapist: Well, maybe you keep thinking that bowling isn't going to solve the problems in your life. You're right, but it's a beginning.

Ascertaining the Meaning of an Event:

Sometimes, skillful attempts by the therapist to elicit automatic thoughts are not successful. Then, the therapist should attempt to discern, through questioning, the specific meaning for the patient of the event that preceded the emotional response. For example, one patient began to cry whenever he had an argument with his girlfriend. It was not possible to identify a specific automatic thought. However, after the therapist asked a series of questions to probe the meaning of the event, it became obvious that the patient had always associated any type of argument or fight with the end of a relationship. It was this meaning, embedded in his view of the event that preceded his crying.

Desirable Therapist Strategies for Identifying Underlying Assumptions:

We often observe general patterns that seem to underlie patients' automatic thoughts. These patterns, or regularities, act as a set of rules that guide the way a patient reacts to many different situations. We refer to these rules as assumptions. These assumptions may determine for example, what patients consider "right" or "wrong" in judging themselves and other people.

Although patients can often readily identify their automatic thoughts, their underlying assumptions are far less accessible. Most people are unaware of their "rulebooks." Typical unarticulated assumptions include:

1. In order to be happy, I have to be successful in whatever I undertake.
2. I can't live without love.

When these rules are framed in absolute terms, are nonrealistic, or are used inappropriately or excessively, they often lead to disturbances like depression, anxiety, and paranoia. We label rules that lead to such problems as "maladaptive."

One of the major goals of cognitive therapy, especially in the later stages of treatment, is to help patients identify and challenge the maladaptive assumptions that affect their ability to avoid future depressions.

In order to identify these maladaptive assumptions, the therapist can listen closely for themes that seem to cut across several different situations or problem areas. The therapist can then list several related automatic thoughts that the patient has already expressed on different occasions, and ask the patient to **abstract the general "rule" that connects the automatic thoughts**. If the patient cannot do this, the therapist can suggest a plausible assumption, list the thoughts that seem to follow from it, and then ask the patient: if the assumption "rings true." The therapist should be open to the possibility that the assumption does not fit that patient and then work with the patient to pinpoint a more accurate statement of the underlying "rule."

Special Considerations in Rating:

There are essentially two separate processes incorporated into this category. The first process involves using appropriate techniques to **elicit** automatic thoughts, underlying assumptions, behaviors, etc. from the patient. If the therapist completely fails to elicit them, then the rater should assign a 0. If the therapist uses appropriate techniques to elicit thoughts and behaviors, he/she should be given a rating of at least 2.

The second step in this process is for the therapist to integrate these cognitions and behaviors into a conceptualization of the patient's problem. The conceptualization explains how the particular constellation of cognitions/behaviors are peripheral to the problem -- and therefore should be postponed -- and which are central and should serve as the **focus** of intervention. If the therapist fails to

focus on a particular thought or behavior, the therapist should be rated 2. Or, if the therapist's conceptualization is so far off that the focus seems totally inappropriate, the therapist should be rated 2.

If the therapist selects a relevant cognition/behavior to focus on, but the rater's conceptualization strongly suggests that some other focus would have been more fruitful, the rater should assign a 4. If the therapist's conceptualization and focus seem very promising and "on target", the rater should assign a 6.

Note that for this item the therapist need not intervene at all to receive a high score. The only requirement is that the therapist successfully elicit relevant thoughts/behaviors, conceptualize the problem, and identify important foci.

9. STRATEGY FOR CHANGE

Objective:

After conceptualizing the problem and pinpointing key cognitions and/or behaviors, the therapist should plan a strategy for change. The strategy for change should follow logically from the conceptualization of the problem and should incorporate the most promising cognitive-behavioral interventions chosen for the particular patient and point in treatment.

Background Material:

Cognitive Therapy and The Emotional Disorders, pp. 233-300 (esp. 257-262); *Cognitive Therapy of Depression, pp. 104-271.*

Rationale:

There are so many different therapeutic tactics available to the cognitive therapist that, unless he/she develops an overall strategy for a given case, the therapy may follow an erratic course based on trial-and-error. The therapist may be employing several procedures simultaneously; when this is the case, all of the procedure should fit together as part of a master plan. The strategy for change should follow logically from the conceptualization of the problem discussed in Section 9 ("Focusing in Specific Cognition or Behaviors").

The overall strategy for change generally incorporates techniques drawn from one or more of three intervention categories: testing automatic thoughts, modifying assumptions, and changing behaviors.

Desirable Techniques for Testing Automatic Thoughts:

Once the therapist and patient have identified a key automatic thought, the therapist asks the patient to suspend temporarily his/her conviction that the thought is undeniably true and instead to view the thought as a hypothesis to be tested. The therapist and patient collaborate in gathering data, evaluating evidence, and drawing conclusions.

This experimental method is basic to the application of cognitive therapy. The therapist help patients learn a **process** of thinking that resembles scientific investigation. The therapist demonstrates to the patient that the **perception** of reality is not the same as reality itself. Patients learn to design experiments which will test the validity of their own automatic thoughts. Patients thus learn how to modify the maladaptive thinking so that they can maintain their gains after treatment ends.

There are several techniques for testing the validity of automatic thoughts:

Examining available evidence

The therapist asks the patient to draw on his/her previous experiences to list the evidence supporting and contradicting the hypothesis. After weighing all available evidence, patients frequently reject their automatic thoughts as false, inaccurate, or exaggerated.

Setting up an experiment

The therapist asks the patient to design an experiment to test the hypothesis. Once the experiment has been planned, the patient predicts what the outcome will be, then gathers data. Frequently the data contradicts the patient's prediction, and the patient can reject the automatic thoughts.

Inductive questioning

When the previous two approaches are not appropriate or applicable, the therapist may produce evidence from his/her own experience that contradicts the patient's hypothesis. This evidence is presented in the form of a question which poses a logical dilemma for the patient (e.g., "90% of my patients say they won't get better, yet most of them do improve. Why do you think you are different from them?"). Alternatively, the therapist, through questioning, may point out logical flaws within the patients' own belief system. (e.g., "You say that you have always been a weak person. Yet you also tell me that before you were depressed you got along fine. Do you see any inconsistency in this thinking?")

Operationalizing a negative construct and defining terms

Sometimes, as a step in testing an automatic thought, the therapist and patient have to define in more concrete terms what the patient means by using a particular word or expression. For example, one patient at our clinic kept telling himself, "I'm a coward." To test the thought, the therapist and patient first had to define and give referents of the construct. In this instance, they operationalized "cowardice" as not defending oneself when being attacked. After this criterion had been agreed upon, the therapist and patient examined past evidence to assess whether the label of "coward" was a valid one. This procedure can help the patient recognize the arbitrary nature of his self-appraisals and bring them more in line with common-sense definitions of these negative terms.

Reattribution

One of the most powerful techniques for testing automatic thoughts is "reattribution." When patients unrealistically blame themselves for unpleasant events, the therapist and patient can review the situation to find other factors that may explain what happened other than, or in addition to, the patient's behavior. This technique may also be used to show patients that some of the problems they are having are symptoms of depression (e.g., loss of concentration) and not indications of permanent physiological deterioration.

Generating alternatives

When patients view particular problems as insoluble, the therapist can work with the patient to generate solutions to the problem that had not been considered. Sometimes the patient has already considered a viable solution, but has prematurely rejected it as unworkable or unlikely to be effective.

Desirable Techniques for Modifying Underlying Assumptions:

The cognitive therapist emphasizes **questioning** in the modification of underlying assumptions. We find that the most effective approach is one in which the patient develops evidence against the assumption either alone or in collaboration with the therapist. After an assumption has been identified, the therapist asks the patient a series of questions to demonstrate the contradictions or problems inherent in the assumption.

Another strategy for testing assumptions is for the therapist and patient to generate **lists of the advantages and disadvantages** of changing an assumption. Once the lists have been completed, the therapist and patient can discuss and weigh the competing considerations. A related approach is for the patient to weigh the long-term and short-term utility of the assumptions.

Many assumptions take the form of "shoulds" -- rules about what patients should ideally do in given situations. A behavioral strategy, "**response prevention**" has been adapted as a technique for overcoming these "shoulds." Once the "should" has been identified, the therapist and patient devise an experiment to test what would happen if the patient did **not** obey the rule. The patient makes a prediction about what the result would be, the experiment is carried out, and the results are discussed. Generally, it is desirable to generate a series of graded tasks which violate the "should," so that the patient attempts less threatening changes first. For example, the patient who believes he "should" work all of the time could experiment with gradually increasing the amount of time devoted to leisure pursuits.

Desirable Techniques for Changing Behaviors:

The cognitive therapist also uses a variety of behavioral techniques to help the patient cope better with situations or inter-personal problems. These behavioral techniques are "action-oriented" in the sense that patients practice specific procedures for dealing with concrete situations or for using time more adaptively. In contrast to strictly cognitive techniques, therefore, behavioral techniques focus more on how to act or cope than on how to view or interpret events.

One of the principle goals of behavioral techniques is to modify dysfunctional cognitions. For example, the patient who believes "I can't enjoy anything anymore" often modifies this automatic thought after completing a series of behavioral assignments designed to increase the number and variety of pleasurable activities he/she engages in. Thus behavioral change is often used as evidence to bring about cognitive change.

Behavioral techniques are incorporated throughout the course of treatment, but are usually concentrated during the early stages of therapy. This is especially true with more severely depressed patients who are immobilized, passive, anhedonic, socially withdrawn and have trouble concentrating.

Brief descriptions of behavioral techniques follow below:

Scheduling activities

The therapist uses an activity schedule to help the patient plan activities hour-by-hour during the day. The patient then keeps a record of the activities that were actually engaged in hour-by-hour. Scheduling activities is usually one of the first techniques used with the depressed patient. It often seems to counteract loss of motivation, hopelessness, and excessive rumination.

Mastery and pleasure

One of the goals of activity scheduling is for patients to derive more pleasure and a greater sense of accomplishment on a day-to-day basis. To do this, the patient rates each completed activity for both mastery and pleasure on a scale from 1 to 10. These ratings generally serve to directly contradict patients' beliefs that they cannot enjoy anything and cannot obtain a sense of accomplishment anymore.

Graded task assignment

In order to help some patients initiate activities for mastery and pleasure, the therapist will have to break down an activity into subtasks, ranging from the simplest part of the task to the most complex and taxing. This step-by-step approach permits depressed patients to eventually tackle tasks that originally seemed impossible or overwhelming to them. These graded tasks provide the immediate and unambiguous feedback to patients that they can succeed.

Cognitive rehearsal

Some patients have difficulty carrying out tasks requiring successive steps for completion. Frequently this is because of problems in concentration. "Cognitive rehearsal" refers to the technique of asking the patient to imagine each step leading to the completion of the task. This rehearsal imagery helps patient focus their attention on the task, and also permits the therapist to identify potential obstacles that may make the assignment more difficult for a particular patient.

Self-reliance training

The therapist may have to teach some patients to take increasing responsibility for their day-to-day activities, rather than relying on other people to take care of all their needs. For example, patients may begin by showering, then making their own beds, cleaning the house, cooking their own meals, shopping, etc. This responsibility also includes gaining control over their emotional reactions. Graded task assignments, assertiveness training, and running experiments may all be used as part of self-reliance training.

Role-playing

In the context of cognitive therapy, role-playing may be used to elicit automatic thoughts in specific interpersonal situations; to practice new cognitive responses in social encounters that had previously been problematic for the patient; and to rehearse new behaviors in order to function more effectively with other people. A variation, role-reversal, is often effective in guiding patients to "reality test" how other people would probably view their behavior, and thus allow patients to view themselves more sympathetically. Role-playing can also be used as part of assertiveness training. Role-playing frequently is accompanied by modeling and coaching procedures.

Diversion techniques

Patients can use various forms of diversion of attention to reduce temporarily most forms of painful affect, including dysphoria, anxiety, and anger. Diversion may be accomplished through physical activity, social contact, work, play, or visual imagery.

Special Note to Raters:

In assessing the strategy for change, the rater should be primarily concerned with how appropriate the particular techniques are for the problems presented by the patient in the session being rated. In deciding the **appropriateness** of the techniques, the rater should try to determine whether the techniques seem to be a part of a coherent strategy for change that follows logically from the therapist's conceptualization of the problem. If the rationale for employing the techniques is not clear, or if the rationale seems faulty, the rater should assign a low score to the therapist. If the rationale seems clear and appropriate, the rater should assign a high score.

The rater should not confuse the quality of the **strategy** for change (which is the main concern of this item) with how effectively the techniques are **implemented** (which is assessed in item 10) or whether change **actually occurred** (which is not necessary to receive a high score on any item).

10. APPLICATION OF COGNITIVE-BEHAVIORAL TECHNIQUES

Objective and Rationale:

Once the therapist has planned a strategy for change that incorporates the most appropriate cognitive-behavioral techniques, he/she must apply the techniques skillfully. Even the most promising strategy will fail if executed poorly.

Background Material:

Cognitive Therapy and The Emotional Disorders, pp. 221-225, 229-232, 250-254, 282-299; *Cognitive Therapy of Depression,* pp. 27-32, 67-72, 104-271, 296-298.

Desirable Application of Techniques:

It is extremely difficult to specify how to know whether a technique is being applied skillfully or not. Clearly, rating this item requires a great deal of clinical judgment and experience. Some general criteria

can be outlined. The therapist should be **fluent** in applying the techniques, rather than fumble around and appear unfamiliar with them. The techniques should be presented **articulately**; in language the patient can easily understand. The techniques should be applied **systematically**, so that there is usually a beginning (introduction, statement of problem, rationale), middle (discussion of possible solutions or change), and end (summary of conclusions, relevant homework assignment). The therapist should be **sensitive** to whether the patient is actually involved in the change process, or merely "going through the motions" out of compliance. The therapist should be **resourceful** in presenting ideas to the patient in such a way that the patient can begin to superimpose the therapist's conflicting views. The therapist needs to anticipate problems the patient may have in changing perspectives or behaviors outside the session. Finally, the therapist should collaborate with the patient rather than debate, cross-examine, or high-pressure him/her.

Example of a Desirable Application:

In the abbreviated example below, the therapist sets up an experiment to test the automatic thought, "I can't concentrate on anything anymore."

Patient: I can't concentrate on anything anymore.

Therapist: How could you test that out?

Patient: I guess I could try reading something.

Therapist: Here's a newspaper. What section do you usually read?

Patient: I used to enjoy the sports section.

Therapist: Here's an article on the Penn basketball game last night. How long do you think you'll be able to concentrate on it?

Patient: I doubt I could get through the first paragraph.

Therapist: Let's write down your prediction. (*Patient writes "one paragraph."*) Now let's test it out. Keep reading until you can't concentrate anymore. This will give us valuable information.

Patient: (*Reads the entire article*.) I'm finished.

Therapist: How far did you get?

Patient: I finished it.

Therapist: Let's write down the results of the experiment. (*Patient writes "eight paragraphs."*) You said before that you couldn't concentrate on anything. Do you still believe that?

Patient: Well, my concentration's not as good as it used to be.

Therapist: That's probably true. However, you have retained some ability. Now let's see if we can improve your concentration.

It is important that the therapist remained neutral regarding the patient's initial prediction and did not assume automatically that the patient's belief was inaccurate or distorted. In some instances, the patient will be correct.

Special Note to Raters:

In assessing how skillfully the therapist applied cognitive-behavioral techniques, the rater must try to ignore whether the techniques are appropriate for the patient's problem (since this is assessed in item 9) and also whether the techniques seem to be working. Sometimes a therapist will apply techniques very

skillfully, yet a particular patient may be extremely rigid or unyielding and does not respond. In such cases, the therapist's flexibility, ingenuity, and patience may justify a high score on this item, even though the patient does not change.

It should also be pointed out that this item refers to the application of techniques designed to **modify** thoughts, assumptions, and behaviors (as outlined in item 9), not to techniques designed primarily to **elicit** cognitions (since the "eliciting" techniques are assessed in item 8).

11. HOMEWORK

Objective:

The therapist assigns homework "custom-tailored" to help the patient test hypotheses, incorporate new perspectives, or experiment with new behavior outside the therapy session. The therapist should also review homework from the previous session, explain the rationale for new assignments, and elicit the patient's reaction to the homework.

Background Material:

Cognitive Therapv of Depression, pp. 272-294.

Rationale:

The systematic completion of homework is of crucial importance in cognitive therapy. Unless patients can apply the concepts learned in the therapy sessions to their lives outside, there will be no progress. Homework, therefore promotes transfer of learning. It also provides a structure for helping patients gather data and test hypotheses, thereby modifying maladaptive cognitions so they are more consistent with reality. Homework thus encourages patients to **concretize** the abstract concepts and insights that have traditionally been the province of psychotherapy, making psychotherapy a more active, involving process. Finally, homework encourages self-control rather than reliance on the therapist, and therefore is important in assuring that the improvement is maintained after termination of treatment.

Desirable Therapist Strategies:

Providing Rationale:

The therapist must stress the importance of homework in treatment. This can be accomplished by explaining the benefits to be derived from each assignment in detail, and periodically reminding patients of how vital these benefits will be in helping the patient improve.

Assigning Homework:

The therapist tailors the assignment to the individual patient. Ideally, it should follow logically from the problems discussed during the session. The assignment should be clear and very specific, and should be written in duplicate (one copy for the patient and one copy for the therapist), usually near the end of the session. Some typical homework assignments include asking patients to:

a. Keep a Daily Record of Dysfunctional Thoughts, with rational responses;
b. Schedule activities;
c. Rate mastery and pleasure;
d. Review a list of the main points made during the session;

e. Read a book or article relevant to the patient's problem;

f. Count automatic thoughts using a wrist counter;

g. Listen to or view a tape of the therapy session;

h. Write an autobiographical sketch;

i. Fill out questionnaires like the Dysfunctional Attitude Scale or the Depression Inventory;

j. Graph or chart hour-by-hour mood changes like anxiety, sadness, or anger;

k. Practice coping techniques like distraction or relaxation; and

l. Try out new behaviors that the patient may have difficulty with (e.g., assertiveness, meeting strangers).

Eliciting Reactions and Possible Difficulties:

It is usually desirable for the therapist to ask patients for their reactions to assignments ("Does it sound useful?" "Does it seem manageable?" "Is the assignment clear?"). It is often helpful for the therapist to suggest that the patient visualize carrying out the assignment to identify any obstacles that might arise. Finally, as therapy progresses, the patient should play an increasing role in suggesting and designing homework assignments.

Reviewing Previous Homework:

Unless the therapist routinely reviews homework assigned from the previous week, the patient may come to believe that there is no need to complete the assignments carefully. Near the beginning of each session, the therapist and patient should discuss each assignment, and the therapist should summarize conclusions derived or progress made.
